# (Dimethyl sulfoxide-κ*O*)(2-formyl­phenolato-κ^2^ *O*,*O*′)[2-(2-oxidobenzyl­idene­amino)­phenolato-κ^3^ *O*,*N*,*O*′]manganese(III)

**DOI:** 10.1107/S1600536811015935

**Published:** 2011-05-07

**Authors:** Inba Raja, Kong Mun Lo, Seik Weng Ng

**Affiliations:** a Department of Chemistry, University of Malaya, 50603 Kuala Lumpur, Malaysia

## Abstract

The O-C_6_H_4_-CH=N–C_6_H_4_-O dianion of the title compound, [Mn(C_13_H_9_NO_2_)(C_7_H_5_O_2_)(C_2_H_6_OS)], acts as an *O*,*N*,*O*′-chelate to bind to the Mn^III^ atom, and the three atoms constitute three points [O—Mn—O = 174.43 (11)°] of an octa­hedron around the metal atom. The azomethine linkage is disordered over two positions in a 0.657 (13):0.343 (13) ratio. The deprotonated salicyldehyde anion acts as an *O*,*O*′-chelate; the sixth coordination site is represented by the O atom of the dimethyl sulfoxide mol­ecule. The crystal studied was a non-merohedral twin with a minor twin component of 14.2 (3)%.

## Related literature

For related Mn^III^ structures, see: Asada *et al.* (1999 ▶, 2002 ▶); Nakamura *et al.* (1999 ▶, 2001 ▶).
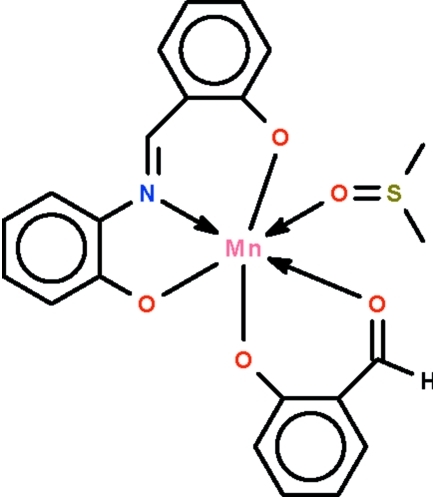


## Experimental

### 

#### Crystal data

[Mn(C_13_H_9_NO_2_)(C_7_H_5_O_2_)(C_2_H_6_OS)]
*M* *_r_* = 465.39
Monoclinic, 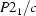

*a* = 12.1342 (3) Å
*b* = 20.4224 (5) Å
*c* = 8.1812 (2) Å
β = 94.692 (2)°
*V* = 2020.59 (9) Å^3^
*Z* = 4
Mo *K*α radiation
μ = 0.79 mm^−1^
*T* = 100 K
0.15 × 0.10 × 0.10 mm

#### Data collection

Bruker SMART APEX diffractometer
Absorption correction: multi-scan (*TWINABS*; Bruker, 2009 ▶) *T*_min_ = 0.891, *T*_max_ = 0.925
39957 measured reflections
5025 independent reflections
3528 reflections with *I* > 2σ(*I*)
*R*_int_ = 0.091

#### Refinement

*R*[*F* ^2^ > 2σ(*F* ^2^)] = 0.058
*wR*(*F* ^2^) = 0.129
*S* = 1.03
5025 reflections
293 parameters
H-atom parameters constrained
Δρ_max_ = 0.60 e Å^−3^
Δρ_min_ = −0.49 e Å^−3^

### 

Data collection: *APEX2* (Bruker, 2009 ▶); cell refinement: *SAINT* (Bruker, 2009 ▶); data reduction: *SAINT*; program(s) used to solve structure: *SHELXS97* (Sheldrick, 2008 ▶); program(s) used to refine structure: *SHELXL97* (Sheldrick, 2008 ▶); molecular graphics: *X-SEED* (Barbour, 2001 ▶); software used to prepare material for publication: *publCIF* (Westrip, 2010 ▶).

## Supplementary Material

Crystal structure: contains datablocks global, I. DOI: 10.1107/S1600536811015935/hg5028sup1.cif

Structure factors: contains datablocks I. DOI: 10.1107/S1600536811015935/hg5028Isup2.hkl

Additional supplementary materials:  crystallographic information; 3D view; checkCIF report

## Figures and Tables

**Fig. 1.**
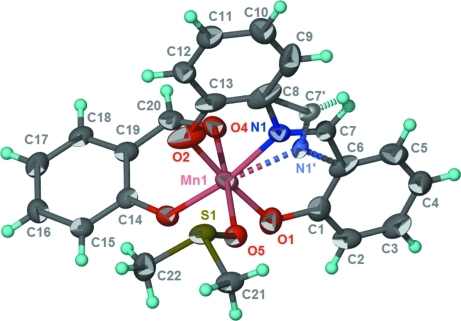
Thermal ellipsoid plot (Barbour, 2001) of Mn(C13H9NO2)(C7H5O3)(C2H6OS) at the 70% probability level; hydrogen atoms are drawn as spheres of arbitrary radius. The disordered azomethine linkage is shown in two conformations.

## supplementary crystallographic information

### Comment

The deprotonated anion of the Schiff base derived by condensing salicylaldehyde and 2-aminophenol, (O–C_6_H_4_–CH═N–C_6_H_4_O)^2-^, behaves as a terdentate *O*,*N*,*O*' chelate to a number of metal ions. For the manganese ion in particular, the crystal structures of a small number of manganese(III) [2-(2-oxidobenzylideneamino)phenolates have been reported (Asada *et al.*, 1999; Asada *et al.*, 2002; Nakamura *et al.*, 1999; Nakamura *et al.*, 2001). In the present study, the reaction of the Schiff base with an mangasese(III) salt resulted in the cleavage of the azomethine linkage of one half of the molar quantity of the organic reactant. The O–C_6_H_4_–CH═N–C_6_H_4_–O dianion of Mn(C_13_H_9_NO_2_)(C_7_H_5_O_3_)(DMSO) (Scheme I) acts as an *O*,*N*,*O*'-chelates to bind to the Mn^III^ atom, and the three atoms constitute three points O–Mn–O 174.4 (1) °] of the octahedron around the metal atom. The azomethine linkage is disordered over two positions in a 66 (1): 34 ratio. The deprotonated salicyldehyde anion acts as an *O*,*O*'-chelate; the sixth coordination site is represented by the O atom of the DMSO molecule (Fig. 1).

### Experimental

2-(Salicylaldimino)phenol was prepared from the condensation reaction of salicyaldehyde and 2-aminophenol. The Schiff base (0.22 g, 1 mmol) and manganese(III) acetate dihydrate (0.23 g, 1 mmol) along with ethanol (100 ml) were heated for an hour until the reactants dissolved completely. A few drops of DMSO were then added. The dark blue solution was then filtered and the solvent allowed to evporate over a few days.

### Refinement

Carbon-bound H-atoms were placed in calculated positions (C—H 0.95 to 0.98 Å) and were included in the refinement in the riding model approximation, with *U*(H) set to 1.2–1.5 times *U*_eq_(C).

The diffraction intensities were separated into two domains and then integrated simultaneously by using *TWINABS* (Bruker, 2009). The '*HKLF 4*' diffraction indices were used for solution whereas the '*HKLF 5*' ones were used for refinement.

There is no excess electron density near the O atom of the salicyldehyde group, which confirms the tripositive oxidation state of Mn.

The azomethine –C═N– linkage is disordered over two positions in a 66 (1): 34 ratio.

### Crystal data

**Table:**

[Mn(C_13_H_9_NO_2_)(C_7_H_5_O_2_)(C_2_H_6_OS)]	*F*(000) = 960
*M**_r_* = 465.39	*D*_x_ = 1.530 Mg m^−^^3^
Monoclinic, *P*2_1_/*c*	Mo *K*α radiation, λ = 0.71073 Å
Hall symbol: -P 2ybc	Cell parameters from 2199 reflections
*a* = 12.1342 (3) Å	θ = 2.7–20.7°
*b* = 20.4224 (5) Å	µ = 0.79 mm^−^^1^
*c* = 8.1812 (2) Å	*T* = 100 K
β = 94.692 (2)°	Prism, blue
*V* = 2020.59 (9) Å^3^	0.15 × 0.10 × 0.10 mm
*Z* = 4	

### Data collection

**Table:**

Bruker SMART APEX diffractometer	5025 independent reflections
Radiation source: fine-focus sealed tube	3528 reflections with *I* > 2σ(*I*)
graphite	*R*_int_ = 0.091
ω scans	θ_max_ = 28.3°, θ_min_ = 2.0°
Absorption correction: multi-scan (TWINABS; Bruker, 2009)	*h* = −16→16
*T*_min_ = 0.891, *T*_max_ = 0.925	*k* = 0→27
39957 measured reflections	*l* = 0→10

### Refinement

**Table:**

Refinement on *F*^2^	Primary atom site location: structure-invariant direct methods
Least-squares matrix: full	Secondary atom site location: difference Fourier map
*R*[*F*^2^ > 2σ(*F*^2^)] = 0.058	Hydrogen site location: inferred from neighbouring sites
*wR*(*F*^2^) = 0.129	H-atom parameters constrained
*S* = 1.03	*w* = 1/[σ^2^(*F*_o_^2^) + (0.0473*P*)^2^ + 1.4711*P*] where *P* = (*F*_o_^2^ + 2*F*_c_^2^)/3
5025 reflections	(Δ/σ)_max_ = 0.001
293 parameters	Δρ_max_ = 0.60 e Å^−^^3^
0 restraints	Δρ_min_ = −0.49 e Å^−^^3^

### Fractional atomic coordinates and isotropic or equivalent isotropic displacement parameters (Å^2^)

**Table:**

	*x*	*y*	*z*	*U*_iso_*/*U*_eq_	Occ. (<1)
Mn1	0.28456 (4)	0.58739 (2)	0.78780 (6)	0.02681 (15)	
S1	0.32300 (7)	0.52236 (4)	1.15078 (9)	0.0296 (2)	
O1	0.4174 (2)	0.61593 (11)	0.7087 (3)	0.0386 (6)	
O2	0.1485 (2)	0.56551 (14)	0.8733 (3)	0.0461 (7)	
O3	0.30142 (17)	0.50104 (10)	0.7054 (2)	0.0260 (5)	
O4	0.19393 (18)	0.60934 (10)	0.5404 (3)	0.0303 (5)	
O5	0.37558 (18)	0.56099 (10)	1.0187 (3)	0.0285 (5)	
N1'	0.2928 (8)	0.6891 (5)	0.8183 (10)	0.018 (3)	0.343 (13)
N1	0.2358 (4)	0.6733 (3)	0.8700 (5)	0.0224 (16)	0.657 (13)
C1	0.4451 (3)	0.67812 (16)	0.6932 (4)	0.0302 (7)	
C2	0.5410 (3)	0.69431 (17)	0.6183 (4)	0.0311 (7)	
H2	0.5868	0.6605	0.5815	0.037*	
C3	0.5696 (3)	0.75874 (18)	0.5973 (4)	0.0361 (8)	
H3	0.6352	0.7688	0.5467	0.043*	
C4	0.5042 (3)	0.80926 (17)	0.6489 (4)	0.0379 (8)	
H4	0.5240	0.8536	0.6321	0.045*	
C5	0.4111 (3)	0.79438 (17)	0.7238 (4)	0.0371 (8)	
H5	0.3664	0.8289	0.7595	0.045*	
C6	0.3802 (3)	0.72972 (17)	0.7493 (4)	0.0308 (7)	
C7'	0.2227 (8)	0.7190 (6)	0.9041 (11)	0.020 (3)	0.343 (13)
H7'	0.2259	0.7646	0.9260	0.024*	0.343 (13)
C7	0.2828 (5)	0.7279 (4)	0.8393 (7)	0.0250 (17)	0.657 (13)
H7	0.2529	0.7676	0.8766	0.030*	0.657 (13)
C8	0.1373 (3)	0.6757 (2)	0.9648 (4)	0.0398 (9)	
C9	0.0843 (3)	0.72355 (18)	1.0517 (4)	0.0451 (10)	
H9	0.1126	0.7669	1.0577	0.054*	
C10	−0.0087 (3)	0.70801 (17)	1.1286 (4)	0.0379 (8)	
H10	−0.0445	0.7404	1.1888	0.046*	
C11	−0.0501 (3)	0.64453 (16)	1.1177 (4)	0.0301 (7)	
H11	−0.1149	0.6338	1.1695	0.036*	
C12	0.0021 (3)	0.59713 (15)	1.0324 (4)	0.0276 (7)	
H12	−0.0275	0.5541	1.0259	0.033*	
C13	0.0974 (3)	0.61137 (18)	0.9557 (4)	0.0328 (8)	
C14	0.2369 (2)	0.46740 (14)	0.5998 (3)	0.0221 (6)	
C15	0.2486 (3)	0.39870 (15)	0.5978 (4)	0.0254 (6)	
H15	0.3033	0.3785	0.6708	0.030*	
C16	0.1821 (3)	0.36033 (15)	0.4920 (4)	0.0290 (7)	
H16	0.1905	0.3141	0.4952	0.035*	
C17	0.1030 (3)	0.38830 (16)	0.3805 (4)	0.0323 (7)	
H17	0.0570	0.3616	0.3087	0.039*	
C18	0.0928 (3)	0.45473 (15)	0.3762 (4)	0.0275 (7)	
H18	0.0396	0.4739	0.2991	0.033*	
C19	0.1585 (2)	0.49587 (15)	0.4825 (4)	0.0254 (6)	
C20	0.1448 (3)	0.56547 (15)	0.4608 (4)	0.0275 (7)	
H20	0.0919	0.5791	0.3755	0.033*	
C21	0.4254 (3)	0.52192 (16)	1.3198 (4)	0.0309 (7)	
H21A	0.4975	0.5112	1.2809	0.046*	
H21B	0.4061	0.4891	1.4000	0.046*	
H21C	0.4290	0.5652	1.3714	0.046*	
C22	0.3339 (3)	0.43848 (16)	1.0928 (4)	0.0366 (8)	
H22A	0.2918	0.4313	0.9869	0.055*	
H22B	0.3040	0.4106	1.1761	0.055*	
H22C	0.4117	0.4275	1.0837	0.055*	

### Atomic displacement parameters (Å^2^)

**Table:**

	*U*^11^	*U*^22^	*U*^33^	*U*^12^	*U*^13^	*U*^23^
Mn1	0.0292 (3)	0.0240 (3)	0.0256 (2)	0.0075 (2)	−0.00755 (19)	−0.00551 (19)
S1	0.0237 (4)	0.0383 (5)	0.0260 (4)	0.0052 (3)	−0.0018 (3)	0.0021 (3)
O1	0.0367 (14)	0.0310 (13)	0.0459 (14)	−0.0077 (10)	−0.0096 (11)	0.0101 (11)
O2	0.0291 (13)	0.0813 (19)	0.0271 (12)	0.0156 (13)	−0.0029 (10)	−0.0159 (12)
O3	0.0278 (12)	0.0252 (11)	0.0238 (10)	0.0042 (9)	−0.0050 (9)	−0.0033 (9)
O4	0.0339 (13)	0.0264 (11)	0.0289 (11)	0.0063 (9)	−0.0068 (10)	−0.0033 (9)
O5	0.0288 (12)	0.0290 (12)	0.0263 (11)	−0.0001 (9)	−0.0053 (9)	−0.0003 (9)
N1'	0.018 (5)	0.020 (6)	0.017 (4)	−0.005 (4)	0.000 (3)	−0.002 (3)
N1	0.020 (3)	0.024 (3)	0.022 (2)	0.0037 (19)	−0.0026 (18)	0.0012 (18)
C1	0.0342 (19)	0.0320 (18)	0.0221 (15)	−0.0041 (14)	−0.0114 (13)	0.0061 (13)
C2	0.0267 (17)	0.041 (2)	0.0258 (15)	0.0085 (14)	0.0014 (13)	0.0022 (14)
C3	0.0267 (17)	0.052 (2)	0.0297 (17)	−0.0045 (16)	0.0018 (14)	0.0147 (15)
C4	0.034 (2)	0.0310 (19)	0.047 (2)	−0.0034 (15)	−0.0108 (16)	0.0073 (15)
C5	0.0338 (19)	0.040 (2)	0.0355 (18)	0.0106 (16)	−0.0096 (15)	−0.0066 (15)
C6	0.0201 (15)	0.052 (2)	0.0190 (14)	−0.0005 (14)	−0.0039 (12)	0.0046 (14)
C7'	0.015 (5)	0.028 (7)	0.017 (4)	0.000 (4)	0.001 (4)	−0.002 (4)
C7	0.026 (3)	0.023 (3)	0.025 (3)	0.001 (2)	−0.008 (2)	−0.003 (2)
C8	0.0242 (18)	0.074 (3)	0.0207 (15)	−0.0046 (17)	−0.0028 (13)	0.0137 (16)
C9	0.053 (2)	0.037 (2)	0.042 (2)	−0.0202 (18)	−0.0217 (18)	0.0140 (17)
C10	0.044 (2)	0.0325 (19)	0.0352 (18)	0.0054 (16)	−0.0078 (16)	−0.0029 (15)
C11	0.0250 (17)	0.0375 (19)	0.0279 (16)	0.0003 (14)	0.0034 (13)	0.0034 (14)
C12	0.0290 (17)	0.0267 (17)	0.0265 (15)	0.0031 (13)	−0.0011 (13)	0.0006 (13)
C13	0.0226 (16)	0.054 (2)	0.0206 (15)	0.0101 (15)	−0.0063 (12)	0.0003 (14)
C14	0.0194 (15)	0.0271 (16)	0.0202 (13)	−0.0017 (12)	0.0052 (11)	0.0007 (12)
C15	0.0221 (15)	0.0292 (17)	0.0250 (15)	0.0023 (12)	0.0021 (12)	−0.0003 (12)
C16	0.0313 (17)	0.0251 (16)	0.0308 (16)	−0.0016 (13)	0.0031 (14)	−0.0017 (13)
C17	0.0288 (18)	0.0367 (19)	0.0304 (17)	−0.0054 (14)	−0.0039 (14)	−0.0052 (14)
C18	0.0204 (16)	0.0353 (18)	0.0264 (15)	0.0007 (13)	−0.0005 (12)	−0.0009 (13)
C19	0.0228 (16)	0.0305 (17)	0.0226 (14)	0.0031 (13)	−0.0001 (12)	0.0007 (12)
C20	0.0254 (17)	0.0318 (17)	0.0246 (15)	0.0061 (13)	−0.0019 (13)	−0.0001 (13)
C21	0.0265 (17)	0.0408 (19)	0.0245 (15)	0.0059 (14)	−0.0031 (13)	0.0011 (14)
C22	0.042 (2)	0.0310 (18)	0.0348 (18)	−0.0077 (15)	−0.0090 (15)	0.0037 (14)

### Geometric parameters (Å, °)

**Table:**

Mn1—O1	1.878 (2)	C7'—H7'	0.9500
Mn1—O2	1.899 (3)	C7—H7	0.9500
Mn1—O3	1.905 (2)	C8—C9	1.396 (5)
Mn1—N1	1.986 (5)	C8—C13	1.401 (5)
Mn1—N1'	2.094 (11)	C9—C10	1.374 (5)
Mn1—O5	2.177 (2)	C9—H9	0.9500
Mn1—O4	2.267 (2)	C10—C11	1.390 (5)
S1—O5	1.520 (2)	C10—H10	0.9500
S1—C21	1.782 (3)	C11—C12	1.377 (4)
S1—C22	1.785 (3)	C11—H11	0.9500
O1—C1	1.322 (4)	C12—C13	1.391 (5)
O2—C13	1.336 (4)	C12—H12	0.9500
O3—C14	1.312 (3)	C14—C15	1.410 (4)
O4—C20	1.233 (4)	C14—C19	1.419 (4)
N1'—C7'	1.299 (19)	C15—C16	1.378 (4)
N1'—C6	1.492 (11)	C15—H15	0.9500
N1—C7	1.285 (11)	C16—C17	1.391 (4)
N1—C8	1.478 (6)	C16—H16	0.9500
C1—C2	1.399 (4)	C17—C18	1.362 (5)
C1—C6	1.414 (5)	C17—H17	0.9500
C2—C3	1.375 (5)	C18—C19	1.408 (4)
C2—H2	0.9500	C18—H18	0.9500
C3—C4	1.388 (5)	C19—C20	1.440 (4)
C3—H3	0.9500	C20—H20	0.9500
C4—C5	1.362 (5)	C21—H21A	0.9800
C4—H4	0.9500	C21—H21B	0.9800
C5—C6	1.393 (5)	C21—H21C	0.9800
C5—H5	0.9500	C22—H22A	0.9800
C6—C7	1.443 (7)	C22—H22B	0.9800
C7'—C8	1.478 (11)	C22—H22C	0.9800
			
O1—Mn1—O2	175.43 (11)	C8—C7'—H7'	122.9
O1—Mn1—O3	92.60 (10)	N1—C7—C6	121.1 (7)
O2—Mn1—O3	91.96 (11)	N1—C7—H7	119.4
O1—Mn1—N1	97.47 (19)	C6—C7—H7	119.4
O2—Mn1—N1	77.96 (19)	C9—C8—C13	120.8 (3)
O3—Mn1—N1	168.85 (17)	C9—C8—C7'	96.8 (5)
O1—Mn1—N1'	72.5 (3)	C13—C8—C7'	142.4 (6)
O2—Mn1—N1'	103.0 (3)	C9—C8—N1	135.8 (4)
O3—Mn1—N1'	162.8 (3)	C13—C8—N1	103.4 (4)
N1—Mn1—N1'	25.3 (2)	C10—C9—C8	120.1 (3)
O1—Mn1—O5	89.25 (9)	C10—C9—H9	119.9
O2—Mn1—O5	90.97 (9)	C8—C9—H9	119.9
O3—Mn1—O5	90.93 (8)	C9—C10—C11	119.4 (3)
N1—Mn1—O5	93.96 (12)	C9—C10—H10	120.3
N1'—Mn1—O5	97.3 (2)	C11—C10—H10	120.3
O1—Mn1—O4	89.92 (9)	C12—C11—C10	120.7 (3)
O2—Mn1—O4	90.13 (9)	C12—C11—H11	119.7
O3—Mn1—O4	85.72 (8)	C10—C11—H11	119.7
N1—Mn1—O4	89.50 (12)	C11—C12—C13	121.0 (3)
N1'—Mn1—O4	85.7 (2)	C11—C12—H12	119.5
O5—Mn1—O4	176.51 (8)	C13—C12—H12	119.5
O5—S1—C21	104.34 (14)	O2—C13—C12	121.2 (3)
O5—S1—C22	105.36 (15)	O2—C13—C8	120.8 (3)
C21—S1—C22	98.00 (16)	C12—C13—C8	118.0 (3)
C1—O1—Mn1	124.3 (2)	O3—C14—C15	118.2 (3)
C13—O2—Mn1	118.3 (2)	O3—C14—C19	124.2 (3)
C14—O3—Mn1	129.96 (19)	C15—C14—C19	117.5 (3)
C20—O4—Mn1	120.4 (2)	C16—C15—C14	121.2 (3)
S1—O5—Mn1	122.20 (12)	C16—C15—H15	119.4
C7'—N1'—C6	117.5 (11)	C14—C15—H15	119.4
C7'—N1'—Mn1	120.2 (9)	C15—C16—C17	121.0 (3)
C6—N1'—Mn1	122.3 (7)	C15—C16—H16	119.5
C7—N1—C8	117.6 (6)	C17—C16—H16	119.5
C7—N1—Mn1	123.4 (5)	C18—C17—C16	118.8 (3)
C8—N1—Mn1	118.9 (4)	C18—C17—H17	120.6
O1—C1—C2	119.7 (3)	C16—C17—H17	120.6
O1—C1—C6	122.1 (3)	C17—C18—C19	122.2 (3)
C2—C1—C6	118.1 (3)	C17—C18—H18	118.9
C3—C2—C1	120.6 (3)	C19—C18—H18	118.9
C3—C2—H2	119.7	C18—C19—C14	119.1 (3)
C1—C2—H2	119.7	C18—C19—C20	117.3 (3)
C2—C3—C4	121.1 (3)	C14—C19—C20	123.5 (3)
C2—C3—H3	119.4	O4—C20—C19	127.3 (3)
C4—C3—H3	119.4	O4—C20—H20	116.4
C5—C4—C3	119.1 (3)	C19—C20—H20	116.4
C5—C4—H4	120.4	S1—C21—H21A	109.5
C3—C4—H4	120.4	S1—C21—H21B	109.5
C4—C5—C6	121.5 (3)	H21A—C21—H21B	109.5
C4—C5—H5	119.3	S1—C21—H21C	109.5
C6—C5—H5	119.3	H21A—C21—H21C	109.5
C5—C6—C1	119.6 (3)	H21B—C21—H21C	109.5
C5—C6—C7	110.1 (4)	S1—C22—H22A	109.5
C1—C6—C7	130.3 (4)	S1—C22—H22B	109.5
C5—C6—N1'	142.3 (5)	H22A—C22—H22B	109.5
C1—C6—N1'	98.1 (5)	S1—C22—H22C	109.5
N1'—C7'—C8	114.2 (11)	H22A—C22—H22C	109.5
N1'—C7'—H7'	122.9	H22B—C22—H22C	109.5
			
O3—Mn1—O1—C1	164.1 (2)	C4—C5—C6—N1'	−179.3 (5)
N1—Mn1—O1—C1	−11.1 (3)	O1—C1—C6—C5	−177.5 (3)
N1'—Mn1—O1—C1	−7.2 (3)	C2—C1—C6—C5	2.1 (4)
O5—Mn1—O1—C1	−105.0 (2)	O1—C1—C6—C7	5.0 (5)
O4—Mn1—O1—C1	78.4 (2)	C2—C1—C6—C7	−175.4 (3)
O3—Mn1—O2—C13	177.8 (2)	O1—C1—C6—N1'	1.2 (4)
N1—Mn1—O2—C13	−7.1 (2)	C2—C1—C6—N1'	−179.2 (4)
N1'—Mn1—O2—C13	−10.9 (3)	C7'—N1'—C6—C5	−11.1 (10)
O5—Mn1—O2—C13	86.8 (2)	Mn1—N1'—C6—C5	171.2 (4)
O4—Mn1—O2—C13	−96.5 (2)	C7'—N1'—C6—C1	170.8 (6)
O1—Mn1—O3—C14	−120.6 (2)	Mn1—N1'—C6—C1	−7.0 (5)
O2—Mn1—O3—C14	59.1 (2)	C7'—N1'—C6—C7	−3.8 (5)
N1—Mn1—O3—C14	34.0 (8)	Mn1—N1'—C6—C7	178.4 (9)
N1'—Mn1—O3—C14	−91.2 (8)	C6—N1'—C7'—C8	178.6 (5)
O5—Mn1—O3—C14	150.1 (2)	Mn1—N1'—C7'—C8	−3.5 (9)
O4—Mn1—O3—C14	−30.8 (2)	C8—N1—C7—C6	179.2 (3)
O1—Mn1—O4—C20	120.4 (2)	Mn1—N1—C7—C6	−3.8 (6)
O2—Mn1—O4—C20	−64.2 (2)	C5—C6—C7—N1	176.5 (4)
O3—Mn1—O4—C20	27.8 (2)	C1—C6—C7—N1	−5.8 (6)
N1—Mn1—O4—C20	−142.1 (3)	N1'—C6—C7—N1	1.2 (5)
N1'—Mn1—O4—C20	−167.2 (4)	N1'—C7'—C8—C9	177.8 (6)
C21—S1—O5—Mn1	−173.72 (14)	N1'—C7'—C8—C13	−5.7 (11)
C22—S1—O5—Mn1	83.62 (17)	N1'—C7'—C8—N1	1.6 (5)
O1—Mn1—O5—S1	−170.71 (15)	C7—N1—C8—C9	−9.2 (6)
O2—Mn1—O5—S1	13.85 (16)	Mn1—N1—C8—C9	173.6 (3)
O3—Mn1—O5—S1	−78.12 (15)	C7—N1—C8—C13	171.5 (4)
N1—Mn1—O5—S1	91.8 (2)	Mn1—N1—C8—C13	−5.6 (3)
N1'—Mn1—O5—S1	117.1 (3)	C7—N1—C8—C7'	−3.9 (6)
O1—Mn1—N1'—C7'	−169.6 (7)	Mn1—N1—C8—C7'	178.9 (6)
O2—Mn1—N1'—C7'	9.9 (6)	C13—C8—C9—C10	−0.5 (5)
O3—Mn1—N1'—C7'	159.4 (6)	C7'—C8—C9—C10	176.9 (4)
N1—Mn1—N1'—C7'	1.1 (5)	N1—C8—C9—C10	−179.7 (4)
O5—Mn1—N1'—C7'	−82.8 (6)	C8—C9—C10—C11	−0.7 (5)
O4—Mn1—N1'—C7'	99.1 (6)	C9—C10—C11—C12	0.8 (5)
O1—Mn1—N1'—C6	8.1 (4)	C10—C11—C12—C13	0.2 (5)
O2—Mn1—N1'—C6	−172.3 (4)	Mn1—O2—C13—C12	−174.9 (2)
O3—Mn1—N1'—C6	−22.9 (11)	Mn1—O2—C13—C8	6.3 (4)
N1—Mn1—N1'—C6	178.8 (9)	C11—C12—C13—O2	179.8 (3)
O5—Mn1—N1'—C6	95.0 (5)	C11—C12—C13—C8	−1.4 (4)
O4—Mn1—N1'—C6	−83.2 (5)	C9—C8—C13—O2	−179.6 (3)
O1—Mn1—N1—C7	10.2 (4)	C7'—C8—C13—O2	4.4 (8)
O2—Mn1—N1—C7	−169.9 (4)	N1—C8—C13—O2	−0.3 (4)
O3—Mn1—N1—C7	−144.2 (7)	C9—C8—C13—C12	1.6 (5)
N1'—Mn1—N1—C7	1.3 (5)	C7'—C8—C13—C12	−174.4 (6)
O5—Mn1—N1—C7	100.0 (4)	N1—C8—C13—C12	−179.1 (3)
O4—Mn1—N1—C7	−79.6 (4)	Mn1—O3—C14—C15	−160.1 (2)
O1—Mn1—N1—C8	−172.8 (3)	Mn1—O3—C14—C19	22.4 (4)
O2—Mn1—N1—C8	7.1 (2)	O3—C14—C15—C16	178.8 (3)
O3—Mn1—N1—C8	32.8 (9)	C19—C14—C15—C16	−3.5 (4)
N1'—Mn1—N1—C8	178.3 (7)	C14—C15—C16—C17	1.6 (5)
O5—Mn1—N1—C8	−83.0 (3)	C15—C16—C17—C18	0.7 (5)
O4—Mn1—N1—C8	97.4 (3)	C16—C17—C18—C19	−0.9 (5)
Mn1—O1—C1—C2	−173.8 (2)	C17—C18—C19—C14	−1.0 (5)
Mn1—O1—C1—C6	5.8 (4)	C17—C18—C19—C20	176.6 (3)
O1—C1—C2—C3	178.4 (3)	O3—C14—C19—C18	−179.3 (3)
C6—C1—C2—C3	−1.3 (4)	C15—C14—C19—C18	3.2 (4)
C1—C2—C3—C4	−0.3 (5)	O3—C14—C19—C20	3.2 (5)
C2—C3—C4—C5	1.1 (5)	C15—C14—C19—C20	−174.3 (3)
C3—C4—C5—C6	−0.2 (5)	Mn1—O4—C20—C19	−17.4 (4)
C4—C5—C6—C1	−1.4 (5)	C18—C19—C20—O4	180.0 (3)
C4—C5—C6—C7	176.6 (3)	C14—C19—C20—O4	−2.5 (5)
